# Outpatient Check-In Using an Online Portal

**DOI:** 10.1089/tmr.2023.0026

**Published:** 2023-10-26

**Authors:** Maikel Immens, Esther Verstraete, Gerdy Klein Bleumink, Ron Pisters

**Affiliations:** ^1^ Department of Neurology, Rijnstate Hospital, Arnhem, the Netherlands.; ^2^ Department of Cardiology, Rijnstate Hospital, Arnhem, the Netherlands.

**Keywords:** digital consultation, outpatient clinic, technological development

## Abstract

**Background:**

Despite ongoing digital and technological developments, incorporation of new developments in outpatient care tends to be slow. Regarding an increasing demand for outpatient care, digitalization of health care carries the potential of a much needed more efficient and patient-oriented system.

**Objective:**

To optimize classic face-to-face outpatient clinic follow-up consultations and evaluate the added value of an upfront digital consult preparation (DCP).

**Methods:**

A cross-sectional observational study was conducted at Rijnstate Hospital (Arnhem, the Netherlands) among all consecutive patients, 18 years or older, who visited the Cardiology (in June 2021) or Neurology (in September 2021) outpatient clinic. All received a DCP survey before their scheduled outpatient clinic appointment, containing three questions regarding their upcoming visit. In addition, the involved health care providers were approached by using a questionnaire to share their experience regarding the DCP. Data concerning the experience of patients and health care providers was anonymous and gathered using Qualtrics.com.

**Results:**

All 25 involved health care providers (12 cardiologists, 13 neurologists) provided feedback. According to the health care providers DCP decreased the workload and improved theirs and patients' preparation. In total, 785 of 1626 (48.3%) patients filled-in the DCP before their appointment within a predetermined period. Only 4% of the patients wanted to change or cancel the consultation. A total of 122 of the 300 (40.1%) patients approached, filled-in a questionnaire to reflect on the DCP. Patients experienced DCP as an improvement of consultation, more time-efficient, increasing patients' and health care providers' preparation, increasing a feeling of acknowledgement and improving co-decision on type of consultation. The DCP did not attribute to co-deciding on treatment.

**Conclusion:**

DCP was perceived as an improvement of the standard outpatient care by both health care providers and patients with automated integration into the electronic patient record being of key importance.

## Introduction

Over the past decade outpatient care has barely seen innovation. Because of our ageing population and the advances in medical science and treatment possibilities, the demand for outpatient clinic has increased. Varied treatments and diagnostics, procedures that once required an inpatient stay can often be replaced by outpatient appointments. Visits to outpatient facilities increased by 14%, between 2005 and 2015, in the United States.^1^ The shift toward the outpatient clinic makes health care more cost-efficient and less time consuming, releasing pressure from clinical wards. However, with an increasing demand for outpatient care, the need for a more efficient and structured way of medical triage and consultation rises.

During the COVID-19 pandemic telehealth usage surged. In April 2020 overall telehealth utilization for outpatient care was 78 times higher than that in February 2020.^2^ Because of COVID-19 pandemic, patients and health care providers acknowledged the efficiency of alternatives for face-to-face consultation. The pandemic has forcefully pushed our health care systems toward a more accessible and cost-efficient way of outpatient care. Even after the pandemic, patients and health care providers are expected to increasingly use technological innovations for outpatient care.

Historically, outpatient appointments—in particular follow-up of chronic intermittent diseases—have been scheduled in advance with a rather arbitrary time window to ensure timely consultation and intervention. A long interval between consultations may render the type of contact inappropriate and some patients may have lost the need for an appointment at that point in time. On the contrary, some patients may need consultation before a scheduled appointment because of unexpected worsening of symptoms or urgent questions.

We hypothesized that digital innovation of outpatient care by using a digital consult preparation (DCP) can create a more “on demand” and personalized structure for outpatient clinics, decrease workload, and increase time-efficiency: a necessary transformation to match the increasing demand. We therefore studied the feasibility of a DCP and explored its potential on workload and time-efficiency.

## Methods

### Recruitment

The study protocol was submitted to the institutional review board who waived the requirement of formal approval being an observational study. A quantitative cross-sectional observational study was conducted at Rijnstate Hospital in Arnhem, the Netherlands. A total of 1626 consecutive patients, 18 years or older, who visited the cardiology (*n* = 931, June 2021) or neurology (*n* = 695, September 2021) outpatient clinic were invited to participate in the DCP survey. They received a single e-mail notification encouraging them to visit their electronic patient portal (EPP) (“Mijn Rijnstate”) and answer a few questions regarding their upcoming visit, without any promise or claim this information will be discussed in the upcoming visit. The DCP survey in the EPP was as follows:

- How have your symptoms evolved for which you have an appointment scheduled?
- Which questions do you want to ask your health care provider?
- Do you feel that the scheduled type of consultation (in person, by phone or video) is still appropriate?

○ If not, what type of consultation would you prefer?

For each question the first three sentences of the patients' response were integrated into the electronic patient record (EPR). Because an automated method to display the response in the EPR in a convenient manner for the health care provider was lacking, we hired secretary staff to copy-paste the information appropriately. Patients indicating a desire to change the type of consultation, based on the third DCP question (i.e. from face-to-face to telemedicine or vice versa). At the neurology department the secretary staff contacted the treating physician to allow shared decision making through evaluation of its medical appropriateness and granted the request accordingly.

The health care providers read the answers during their preparation and addressed them during the consecutive consultation.

**FIG. 1.**
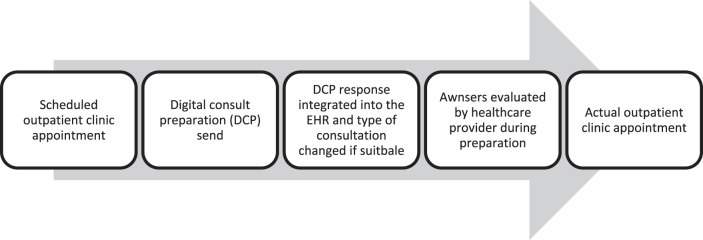
DCP procedure. DCP, digital consult preparation.

To evaluate the DCP from a patient's perspective, evenly divided between cardiology and neurology participants, the first 300 consecutive patients who filled-in the DCP were approached to fill-in a feedback questionnaire (Table 1). The questionnaire was e-mailed, without any reminder, on December 7, 2021. Patients responded between December 7, 2021 and January 5, 2022. There was no financial benefit or other type of compensation offered. Patients were informed that all responses would be processed anonymously. In addition, all cardiologists (*n* = 12) and neurologists (*n* = 13) at Rijnstate Hospital were approached to share their experiences regarding the DCP. Using Qualtrics.com we gathered anonymized data concerning the experiences of patients and health care providers (Tables 1 and 2) and information on the appointments that were cancelled or changed owing to DCP (Table 4). The procedure is given in Figure 1.

**Table 1.** Questions Addressed to Patients

Demographics: sex, age
On a scale of 1 (very negative), 2 (negative), 3 (neutral), 4 (positive) to 5 (very positive), with 0 being “missing” ○ I experience digital consult preparation as … ○ Digital consult preparation is a … addition to my consultation. ○ Digital consult preparation affects my preparation for the consultation in a … way. ○ Digital consult preparation affects the preparation by my health care provider in a … way. ○ Digital consult preparation affects the time me and my health care provider need to arrive at the core of the problem in a … way. ○ Digital consult preparation affects the way I can co-decide about the type of consultation (e.g., phone-/video call, in person, to postpone the appointment) in a … way. ○ Digital consult preparation affects the way my health care provider understands me … ○ Digital consult preparation affects the way I can co-decide about my choice of treatment …
I had the impression that my health care provider has read my upfront general check questionnaire. Agree or disagree.
Digital consult preparation is worth the effort. Agree or disagree.
Have you noticed any advantages concerning digital consult preparation, which is not mentioned above?
Have you noticed any disadvantages concerning digital consult preparation, which is not mentioned above?
Do you have any suggestions to improve digital consult preparation?

The original questionnaire was in Dutch.

Questions addressed to the patients regarding their experience with digital consult preparation.

**Table 2.** Questions Addressed to Health Care Providers

Demographics: sex, age
Medical specialty. Neurologist or cardiologist.
On a scale of 1 (very negative), 2 (negative), 3 (neutral), 4 (positive) to 5 (very positive), with 0 being a “missing” factor. ○ I experience digital consult preparation as … ○ Digital consult preparation affects my workload in a … way. ○ Digital consult preparation affects the time I spend on my consultation in … way. ○ Digital consult preparation affects my preparation before the consult in a … way. ○ Digital consult preparation affects the preparation of my patients in a … way. ○ Digital consult preparation affects the number of actions needed in a … way.
Have you noticed any advantages concerning digital consult preparation, which is not mentioned above?
Have you noticed any disadvantages concerning digital consult preparation, which is not mentioned above?

The original questionnaire was in Dutch.

Questions addressed to the health care providers regarding their experience with digital consult preparation.

**Table 3.** Patient Information

		Missing data
Total responses, *n* (%)	122 (40.7)	—
Male, *n* (%)	70 (61.4)	8 (6.6)
Age, *n* (%)		7 (5.7)
18–30 years	1 (0.9)	
30–50 years	11 (9.6)	
50–70 years	61 (53.0)	
>70 years	42 (36.5)	
I have the impression that my health care providers has read my DCP, *n* (%)	76 (72.4)	17 (13.9)
Digital consult preparation is worth the effort, *n* (%)	89 (83.2)	15 (12.3)

Results of the questionnaire filled-in by the patients regarding the experience of digital consult preparation.

DCP, digital consult preparation.

**Table 4.** Utilization of Digital Consult Preparation

Completed the DCP questionnaire, *n* (%)	785 (48.3)
Male, *n* (%)	419 (53.4)
Mean age (IQR)	63 (18)
Type of consultation is still appropriate, *n* (%)	731 (93.1)
Type of consultation is not appropriate anymore, *n* (%)	32 (4.0)
Would like to switch to an in-hospital appointment, *n* (%)	13 (1.7)
Would like to switch to a (video-) call appointment, *n* (%)	9 (1.1)
Would like to cancel the appointment, *n* (%)	3 (0.4)
Would like to postpone the appointment, *n* (%)	3 (0.4)
Unclear, *n* (%)	4 (0.5)
Data missing, *n* (%)	22 (2.8)

Results of the utilization of the digital consult preparation by patients.

IQR, interquartile range.

### Statistical analysis

Continuous variables are reported as median (interquartile range [IQR]) and categorical variables as number of observed patients (percentage). Patients received eight questions regarding their experience with the DCP. Patients answered using a numerical scale: 0 (not applicable), 1 (very negative), 2 (negative), 3 (neutral), 4 (positive) and 5 (very positive). A mean > 3 indicates a positive response, whereas <3 indicates a negative response to the question, 0 was excluded from the calculation of the median.

## Results

A total of 1626 consecutive patients received a digital invitation, without a reminder, to complete their DCP before their scheduled cardiology (931, 57%) or neurology (695, 43%) outpatient clinic appointment. This led to 785 (48.3%) patients filling-in their DCP.

In total, 13 neurologists and 12 cardiologists participated of which 12 (48%) were men, 8 (32%) were below the age of 40 years and 11 (44%) were above the age of 50 years.

### DCP experience—patient perspective

A total 122 (40.7%) of the 300 DCP participants, randomly invited to provide feedback, responded. One hundred fourteen patients filled-in their sex as either man or woman. Seventy (61.4%) patients were men. Most patients were between the age of 50 and 70 years (53.0%) and only one patient was between the age of 18 and 30 years (0.9%). Results depicted in Table 3.

The question answered most positively was “I experience the digital consult preparation as…” (median = 4.0, IQR = 3.0–5.0). The questions about how the DCP affects: time-efficiency (median = 4.0, IQR = 3.5–4.5), improvement of the consultation (median = 4.0, IQR = 3.5–4.5), patients preparation (median = 4.0, IQR = 3.5–4.5), and health care providers preparation (median = 4.0, IQR = 3.5–4.5) were all answered predominantly positive. The questions on co-decision on type of consultation (median = 3.0, IQR = 2.5–3.5) and acknowledgement of the patient by the health care provider (median = 3.0, IQR = 2.5–3.5) were answered neutrally with a positive tendency. The question if DCP affects the way patients can co-decide about their own treatment (median = 3.0, IQR = 2.0–4.0) was answered neutrally with a negative tendency. With 72.4%, the vast majority of patients had the impression that their health care provider had read their DCP. Furthermore, 83.2% of the patients believe DCP is worth the effort.

Some of the patients who answered one or more questions as “negative” or “very negative” noted specific remarks regarding the DCP. These remarks were: not being able to co-decide, the health care provider not going through the questionnaire together with the patient, other subjects outside the questionnaire were more difficult to address, the DCP felt impersonal and having the feeling that the health care provider did not read the DCP.

Results of the questionnaire send to the patients are given in Table 5.

**Table 5.** Results Patients

Questions for patients	Very negative, ***n*** (%)	Negative, ***n*** (%)	Neutral, ***n*** (%)	Positive, ***n*** (%)	Very positive, ***n*** (%)
I experience digital consult preparation as …	0 (0)	6 (6)	25 (23)	**48 (44)**	30 (28)
Digital consult preparation is a … addition to my consultation	0 (0)	4 (4)	29 (29)	**45 (45)**	22 (22)
Digital consult preparation affects my preparation for the consultation in a … way	3 (3)	4 (4)	33 (32)	**39 (38)**	23 (23)
Digital consult preparation affects the preparation of my health care provider in a … way	1 (1)	5 (5)	34 (34)	**42 (42)**	18 (18)
Digital consult preparation affects the time me and my health care provider need to arrive at the core of the problem …	3 (3)	7 (7)	30 (31)	**39 (40)**	18 (19)
Digital consult preparation affects the way I can co-decide about the type of consultation (e.g., phone-/video call, physical consult, to postpone the appointment) …	5 (5)	8 (8)	**40 (41)**	31 (32)	14 (14)
Digital consult preparation affects the way my health care provider understands me …	6 (6)	11 (11)	**38 (39)**	32 (33)	10 (10)
Digital consult preparation affects the way I can co-decide about my choice of treatment …	7 (7)	11 (12)	**41 (44)**	27 (29)	8 (9)

The most frequently given answers are highlighted in bold.

Results of the questionnaire filled-in by the patients regarding the experience of digital consult preparation.

### DCP experience—health care provider perspective

All health care providers filled-in the questionnaire. Twelve (48%) health care providers were men. The question answered most positively was “I experience the digital consult preparation as…” (median = 4.0, IQR = 3.75–4.25). The question on patients preparation (median = 4.0, IQR = 3.0–5.0) was answered predominantly positive. Other questions answered neutrally with a positive tendency were about the decrease of workload (median = 3.0, IQR = 2.5–3.5) and health care providers preparation (median = 3.0, IQR = 2.5–3.5). The questions regarding time-efficiency (median = 3.0, IQR = 3.0–3.0) and number of actions needed (median = 3.0, IQR = 2.0–4.0) were answered neutrally with a negative tendency.

Results of the questionnaire sent to the health care providers are given in Table 6.

**Table 6.** Results Health Care Providers

Questions for health care providers	Very negative, ***n*** (%)	Negative, ***n*** (%)	Neutral, ***n*** (%)	Positive, ***n*** (%)	Very positive, ***n*** (%)
I experience digital consult preparation as …	0 (0)	0 (0)	6 (24)	**15 (60)**	4 (16)
Digital consult preparation affects my workload in a … way	0 (0)	3 (13)	**11 (46)**	10 (42)	0 (0)
Digital consult preparation affects the time I spend on my consultation in a … way	1 (5)	2 (9)	**14 (64)**	5 (23)	0 (0)
Digital consult preparation affects my preparation before the consult in a … way	1 (4)	1 (4)	**11 (44)**	10 (40)	2 (8)
Digital consult preparation affects the preparation of my patients in a … way	0 (0)	0 (0)	6 (26)	**17 (74)**	0 (0)
Digital consult preparation affects the number of actions needed in a … way	1 (5)	6 (27)	**13 (60)**	2 (9)	0 (0)

The most frequently given answers are highlighted in bold.

Results of the questionnaire filled-in by the health care providers regarding the experience of digital consult preparation.

### DCP—shared decision making in telemedicine

Of the 32 (4.0%) patients who deemed the type of consultation no longer appropriate, 13 preferred a face-to-face consultation instead of the planned phone or video call, whereas 8 preferred consultation by phone and 1 by video call opposed to the scheduled in-person appointment. Furthermore, three patients wanted to postpone and another three patients wanted to completely cancel their planned appointment, creating a total of 15 patients who did not feel the need to visit the hospital at the planned time. Results on utilization of the DCP are given in Table 4.

## Discussion

The cross-sectional ROBOCOP study demonstrated that optimizing classic face-to-face outpatient clinic consultations through an integrated, upfront DCP was worthwhile for the majority of patients and has a (very) positive health care provider experience. The DCP showed the potential to decrease workload for the health care provider and improve patient and health care provider preparation. The number of required actions in the EPR and time-efficiency can be deemed potential hurdles. Although limited, the number of requests to switch from telemedicine to face-to-face consultation or vice versa was balanced.

### Experience

Our results regarding user experience, both from a patient as well as from a health care professional perspective, are very promising, especially in light of the inevitable hurdles associated with pilots, which are described hereunder. Patients experience added value as the majority perceive an improved efficiency of the consultation, which probably relates to the improved preparation by both patient and health care provider. Furthermore, patients feel more understood by their health care professional. In fact, the only aspect in which the DCP failed to be of added value was to increase shared decision making regarding their treatment, which is not surprising considering in its current format the DCP was not designed to address this issue specifically. Of importance is the observation that the majority of patients are under the impression that their health care provider has read the DCP. Although this might seem trivial, when only a sense of futility arises noncompliance or reluctance to fill-out any form is easily created.

Health care professionals have a similar positive experience with the DCP in general. They also report an improved preparation, not just by their patients but also by themselves. Of interest, health care professionals perceive a reduced work load while using the DCP. This is extremely important given the increasing demand for outpatient care and the pitfall of many innovations to increase, instead of decrease the workload.

### Hurdles

We encountered hurdles mostly related to an absent *savvy* integration of the DCP in the EPR. Although we were fortunate that a patient's response was automatically uploaded into the EPR, the actual storage location of this information was not practical. We resorted to a work-around solution where administrative personnel manually copy-pasted the patient response into the work page of the health care provider. As such (1) it was instantly clear whether or not the patient filled-out the DCP and (2) the provided information could be readily incorporated into the actual consultation report.

However, because we were unable to provide this administrative support in all cases, health care providers needed more clicks and perform additional actions now and then. The importance of a savvy integration is underlined by the fact that the only negative feedback from the health care providers was regarding time-efficiency and number of actions needed. At present, the integration has improved and the DCP can easily be loaded onto the work page of the health care provider, which reduces the registration burden.

### Shared decision making: in person versus teleconsultation

Without any prior explanation or additional information patients were allowed to indicate if they felt their mode of consultation was appropriate or not, that is, if in-person versus teleconsultation or vice versa was preferred. The response from the 4% of patients who deemed the mode inappropriate indicates the importance of a hybrid pathway as the number of requests to switch from in-person to teleconsultation was similar to the other way around. However, we feel that the number of patients who would like to change the mode of consultation, postpone or even cancel the scheduled appointment is underestimated. Given the absent explanation of this particular question, which would include expression of medical approval of the decision, one can imagine patients being reluctant to ask for such radical change.

### Future directions

The DCP has the potential to expand to serve a broader range of medical specialties. Furthermore, the DCP will be extended with more disease-specific questions. This allows physicians to dispose of the most up-to-date and relevant information which, among others, facilitates decision making on patient requests to change the mode or timing of the consultation.

According to the evaluation, DCP improved the preparation of the patient and the health care provider. The DCP primed patients and got them thinking about questions they had for the health care provider, about their symptoms and state-of-mind. Thoughtful extension of the DCP potentially allows them to be even better prepared and be instrumental in shared decision making. Taking it one step further, we foresee automated DCP as the follow-up mode. In this scenario outpatient clinic visits or calls are only scheduled if deemed necessary based on the DCP. These developments can only be successful if and when a savvy incorporation of the DCP is realized. This should address the hurdles of (time) inefficiency for health care providers and patients alike.

### Limitations

DCP requires a certain basic digital dexterity. Subsequently, patients lacking the confidence, technical tools, or skills to fill-in a digital questionnaire were not part of our study and results should be interpreted accordingly. Although in our case all specialists participated, same holds true for health care providers. Understanding how to maximize the onboarding and usage of DCP and other telemedicine tools should be one of our main priorities to exploit their full potential.

The feedback questionnaire was send to a fraction of patients who participated in the DCP, which limits its representativeness and is inherently subjective to selection bias. However, the questionnaire was sent out randomly, evenly divided between the different outpatient clinics, to a fair number of DCP participants (roughly half) and the response rate (40%)—without any reminder—was decent. Furthermore, to prevent bias in reporting we informed patients upfront that their responses would be anonymously processed. Unfortunately, we can only speculate as to why 60% of the patients did not respond to the feedback questionnaire.

Given the time between filling-out the DCP and providing their feedback was 3–6 months for patients there is a recall bias. At the same time, the uniqueness of the DCP could mitigate the impact of recall bias.

Finally, a potential pitfall of DCP could be not recognizing, deteriorating, or serious conditions, especially upon deciding to postpone or cancel regular consultation.^3^ Therefore, it is of utmost importance to use electronic replacement of standard care in a responsible way, give good instructions to patients and their family, and monitor its impact on quality of care.

## Conclusion

The DCP is easy to implement in different outpatient clinics, allows for more tailor-made consultations, and has overall been seen as an improvement to the standard outpatient care by both health care providers and patients.

## Abbreviations Used

DCP: digital consultation preparation
EPP: electronic patient portal
EPR: electronic patient record
IQR: interquartile range
